# Detection of H1N1 Influenza Virus in the Bile of a Severe Influenza Mouse Model

**DOI:** 10.1111/irv.70012

**Published:** 2024-10-25

**Authors:** Yan Liu, Jiuyang Xu, Cheng Wei, Yitian Xu, Chen Lyu, Mingzhi Sun, Ying Zheng, Bin Cao

**Affiliations:** ^1^ State Key Laboratory of Respiratory Health and Multimorbidity National Center for Respiratory Medicine China; ^2^ National Clinical Research Center for Respiratory Diseases; Institute of Respiratory Medicine Chinese Academy of Medical Sciences China; ^3^ Department of Pulmonary and Critical Care Medicine, Center of Respiratory Medicine China‐Japan Friendship Hospital, Capital Medical University Beijing China; ^4^ Department of Critical Care Medicine Yantai Affiliated Hospital of Binzhou Medical University Yantai Shandong China; ^5^ Peking University China‐Japan Friendship School of Clinical Medicine Beijing China; ^6^ Peking Union Medical College and Chinese Academy of Medical Sciences Beijing China; ^7^ Tsinghua University School of Medicine Beijing China; ^8^ Department of Pulmonary and Critical Care Medicine China‐Japan Friendship Hospital, Capital Medical University Beijing China

**Keywords:** bile, influenza, liver, mouse model, viral sepsis, viremia

## Abstract

**Aims:**

Influenza virus infection may lead to fatal complications including multi‐organ failure and sepsis. The influenza virus was detected in various extra‐pulmonary organs in autopsy studies during the 2009 pandemic. However, limited research has been conducted on the presence of viral particle or viral components in the peripheral blood.

**Methods and Results:**

We established a mouse model for severe H1N1 influenza. The bile and blood samples were collected over time and inoculated into embryonated chicken eggs. We detected live influenza virus in bile and blood samples in early infection. Immunofluorescence showed influenza viral components in the liver tissue. No live virus was isolated in the bile in mice intragastrically administered with influenza virus, indicating that the virus was spread from the blood stream. Targeted metabolomics analysis of bile acid and liver tissues showed that a secondary bile acid (3‐dehydrocholic acid) was decreased after influenza H1N1 infection. Genes related with fatty acid metabolism and bile secretion pathways were down‐regulated in liver after influenza virus infection.

**Conclusion:**

Our study indicated that influenza virus viremia is present in severe influenza, and that the liver is a target organ for influenza viral sepsis.

## 1 Introduction

Influenza virus leads to annual outbreaks and irregular epidemics, threatening human health. Influenza virus usually infects respiratory tissues and causes self‐limited upper‐respiratory tract symptoms in the general population [1]. However, in some cases, it progresses to pneumonia, multi‐organ failure, and even sepsis, especially in infection with the highly pathogenic avian influenza virus or during global pandemic [2, 3]. The viral RNA and protein have been detected in various extrapulmonary tissues, including heart [4], liver, kidney, and brain [5, 6] in patients with severe influenza. Viremic spread is suspected and detection of viral RNA in blood has been frequently reported. However, recovery of replication‐competent influenza virus from blood has rarely been demonstrated. Only a few clinical cases [2, 7] and animal studies [8] with highly pathogenic avian influenza have reported successful recovery of live virus from blood stream.

In order to study whether influenza viremia is present in infection with non‐pandemic or non‐highly pathogenic avian influenza virus, we utilized an established severe influenza model in C57BL/6 and BALB/c mice with influenza virus strain PR8 and WSN. We collected peripheral blood sample early after influenza infection and cultured for replication‐competent influenza virus. Since some of the circulatory components may be captured by the liver and excreted to the intestinal tract through the bile [9, 10], we also collected the bile sample for viral propagation. We detected live influenza virus in both the bile and blood samples early after influenza infection and found viral components in the liver. Influenza virus infection also induced metabolic changes in liver cells. These results indicate that influenza virus viremia is present in severe influenza, and that the liver is a target organ for influenza viral sepsis.

## 2 Materials and Methods

### 2.1 Virus and Cells

The influenza strain A/H1N1/Puerto Rico/8/34 (PR8) virus was obtained from American Type Culture Collection (ATCC). The influenza virus strain A/H1N1/WSN/1933 (WSN) was kindly provided by Jun Wang (Institut Pasteur of Shanghai, Shanghai, China). The Madin–Darby canine kidney cell line (MDCK) was obtained from ATCC. MDCK cells were cultured using Dulbecco's modified Eagle's medium (DMEM) containing 10% fetal bovine serum (FBS) and 1% penicillin/streptomycin at 37°C in a 5% CO_2_ atmosphere.

### 2.2 Virus Propagation in Embryonated Chicken Eggs

The mouse gallbladder was transferred into a sterile Ethylene Propylene (EP) tube and punctured to obtain bile. Then 200 μL sterile Phosphate buffered saline (PBS) was added, vortexed and centrifuged for several seconds. The supernatant was injected into the allantoic cavity of a 10‐day‐old specific pathogen free (SPF) embryonated chicken egg. The blood was obtained by cardiac puncture and inoculated into embryonated chicken eggs as above. The embryonated eggs were incubated at 37°C for 48 h before viral titration.

### 2.3 Virus Titration by Hemagglutination Assay

The titre of influenza virus in the harvested allantoic fluid was detected using hemagglutination (HA) assay with 1% chicken red blood cells. Fifty microliters of allantoic fluid was diluted at 1:2 using phosphate‐buffered saline in a 96‐well blood coagulation plate. Then, equal volume of 1% chicken red blood cells were added and observed at room temperature for 30–45 min. The highest dilution at which blood coagulation appeared was the HA titre. Allantoic fluid aliquots were placed in a −80°C freezer until further use.

### 2.4 Viral Titration by Plaque Assay

MDCK cells were seeded into 12‐well plates and virus preparations were serially diluted (10‐fold) in DMEM serum‐free medium. Cells were washed twice with PBS and infected with 200 μL dilution for 2 h at 37°C, after which a solid overlay comprising DMEM medium and 1% agarose was added. Forty‐eight hours after infection, cells were fixed with 4% PFA and stained with 0.2% crystal violet, and plaques were enumerated.

### 2.5 Animals

The animal experiments were designed and reported under the ARRIVE guidelines. Eight to 12‐week‐old male wild‐type C57BL/6 and BALB/c mice were purchased from SPF (Beijing) Biotechnology Co. Ltd. All animals were bred and maintained under Beijing Laboratory Animal Research Center Co. Ltd. The C57BL/6 mice were anesthetized with pentobarbital before intranasally (i.n.) inoculation with 1 × 10^6^ plaque‐forming units (PFU) of influenza A/Puerto Rico/8/34 (PR8) virus (*n* = 15). The mice in mock group (*n* = 4) were anesthetized and inoculated with i.n. PBS at the same volume. The mice were randomly assigned to experiment or mock group by cage. Sample size was determined prior to the study [11]. Mice in gavage‐control group (*n* = 6) were gavaged with 1 × 10^4^ PFU PR8 in 50 μL. Mice were then monitored daily for weight loss and survival for 15 days. We monitored general condition and body weight of the mice daily post infection. The mice were euthanized if they have any signs of suffering, defined as weight loss over 20%, weakness or the inability to eat or drink. Kaplan–Meier survival curves were plotted for each group. The blood and bile samples were harvested at the indicated time. After sterilizing the mouse fur with 75% ethanol, the peritoneal cavity was first open and the gallbladder was carefully removed with another clean pair of forceps and scissors. The chest cavity was then opened and blood sample was collected by cardiac puncture using a sterile 1 mL syringe pre‐treated with 2.5 mM EDTA without touching the lungs.

### 2.6 Histology

Paraformaldehyde (PFA)‐fixed and 4‐μm paraffin‐embedded liver sections were stained with periodic acid‐Schiff (PAS), hematoxylin and eosin (HE) for morphological analysis. Frozen tissue sections were used for immunohistochemical analysis and oil red O staining. Gallbladders and liver samples were fixed with 4% PFA overnight, dehydrated in gradient sucrose overnight, and embedded with Tissue‐Tek OCT. Then 10 μm thick OCT‐embedded tissue sections were prepared. Sections were permeabilized with 0.1% Triton X‐100 for 10 min, then blocked with Mouse on Mouse (MOM) Blocking Reagent (Vector Laboratories, USA) that diluted in 1% BSA‐PBS solution to block the endogenous mouse IgG reactivity. Then gallbladder sections were labeled with primary anti‐influenza A virus nucleoprotein (NP) antibody (1:100; mouse anti‐influenza NP, Santa Cruz, USA) overnight in 4 °C in wet box. Alex Flour 488‐rabbit anti mouse secondary antibody (1:1000; Invitrogen, A‐11059, USA) was used. Frozen sections of liver tissues were blocked with Mouse on Mouse (MOM) blocking reagent and 10% goat serum for 1 h. The primary antibodies were diluted with 3%BSA‐PBS solution: influenza A NP antibody‐FITC (1:100; Invitrogen, MA1‐7322, USA), anti‐F480 (1:100; rat anti‐mouse; Invitrogen, 14‐4801‐82, USA), anti‐CD31 rabbit primary antibody (1:300; Servicebio; GB11063‐3, China), incubated in wet box and in dark overnight in 4°C. Secondary antibodies: alexa flour 594‐goat anti‐rat (1:100; Invitrogen, A‐11007, USA), donkey anti‐rabbit highly cross‐adsorbed secondary antibody, alexa fluor 647(1:1000; Invitrogen, A‐31573, USA) were added and incubated for 1 h in room temperature in dark. Following extensive washing, sections were mounted with DAPI‐containing medium (Abcam, ab104139, USA). All slides were scanned and analyzed with Pannoramic scanner (3D Histech Ltd.).

### 2.7 Negative Staining Electron Microscopy

Allantoic fluid from the chicken embryos inoculated with blood and bile samples were observed under negative staining electron microscopy. Sixty microliters 40% formaldehyde solution was added into EP tubes that contained 900 μL allantoic fluid and inactivated for at least 2 h. Then the enriched supernatant was negatively stained on film‐coated grids for examination. Samples were then fixed with 1% osmium tetroxide dehydrated with grade ethanol embedded with PON812 resin. Sections (80 nm) were cut from resin block and stained with uranyl acetate and lead citrate, separately. The negative stained grids and ultrathin sections were observed under transmission electron microscopy.

### 2.8 Bulk RNA Sequencing

Liver samples of mice (24 h post infection) were harvested and total RNA was extracted with TRIzol reagent (Thermo Fisher Scientific, USA). High‐throughput RNA‐sequencing was performed by Novogene Technology (Beijing, China). RNA integrity was assessed using the RNA Nano 6000 Assay Kit of the Bio analyzer 2100 system (Agilent Technologies, CA, USA). Differential expression analysis of two groups was performed using the edgeR R package (3.22.5). The *p* values were adjusted using the Benjamini & Hochberg method. Corrected *p*‐value of 0.05 and absolute fold change of 2 were set as the threshold for significantly differential expression. Differential expression analysis of mock (HC) and PR8 groups was performed using the DESeq2 R package [12, 13] (1.20.0). The resulting *p*‐values were adjusted as above. Genes with an adjusted *p*‐value <0.05 found by DESeq2 were assigned as differentially expressed. Gene Ontology (GO) and Kyoto Encyclopedia of Genes and genomes (KEGG) enrichment analysis of differentially expressed gene were implemented by the clusterProfilter R package [14], in which gene length bias was corrected. The threshold of *p* value was 0.05. The Benjamini–Hochberg procedure was used to adjust the *p* value. Gene set enrichment analysis (GSEA) was performed using clusterProfiler v 4.10.0 and visualized by R package GseaVis v 0.1.0.

### 2.9 Bile Acid Metabolomic

The bile acid targeted metabolomic was performed by Novogene Technology (Beijing, China) using the same lobe of liver for bulk RNA sequencing. An ultra‐high performance liquid chromatography coupled to tandem mass spectrometry (UHPLC–MS/MS) system (ExionLC™ ad UHPLC‐QTRAP 6500+, AB SCIEX Corp., Boston, MA, USA) was used to quantitate bile acids. Data acquisition and instrumental control were performed with Analyst 1.7 software (Sciex, Darmstadt, Germany). The data were analyzed with MultiQuant 3.0.3 (Sciex, Darmstadt, Germany) and Metaboanalyst [15].

## 3 Results

### 3.1 Detection of Influenza Virus in Mouse Bile Early During Intra‐Nasal Infection

**FIGURE 1:**
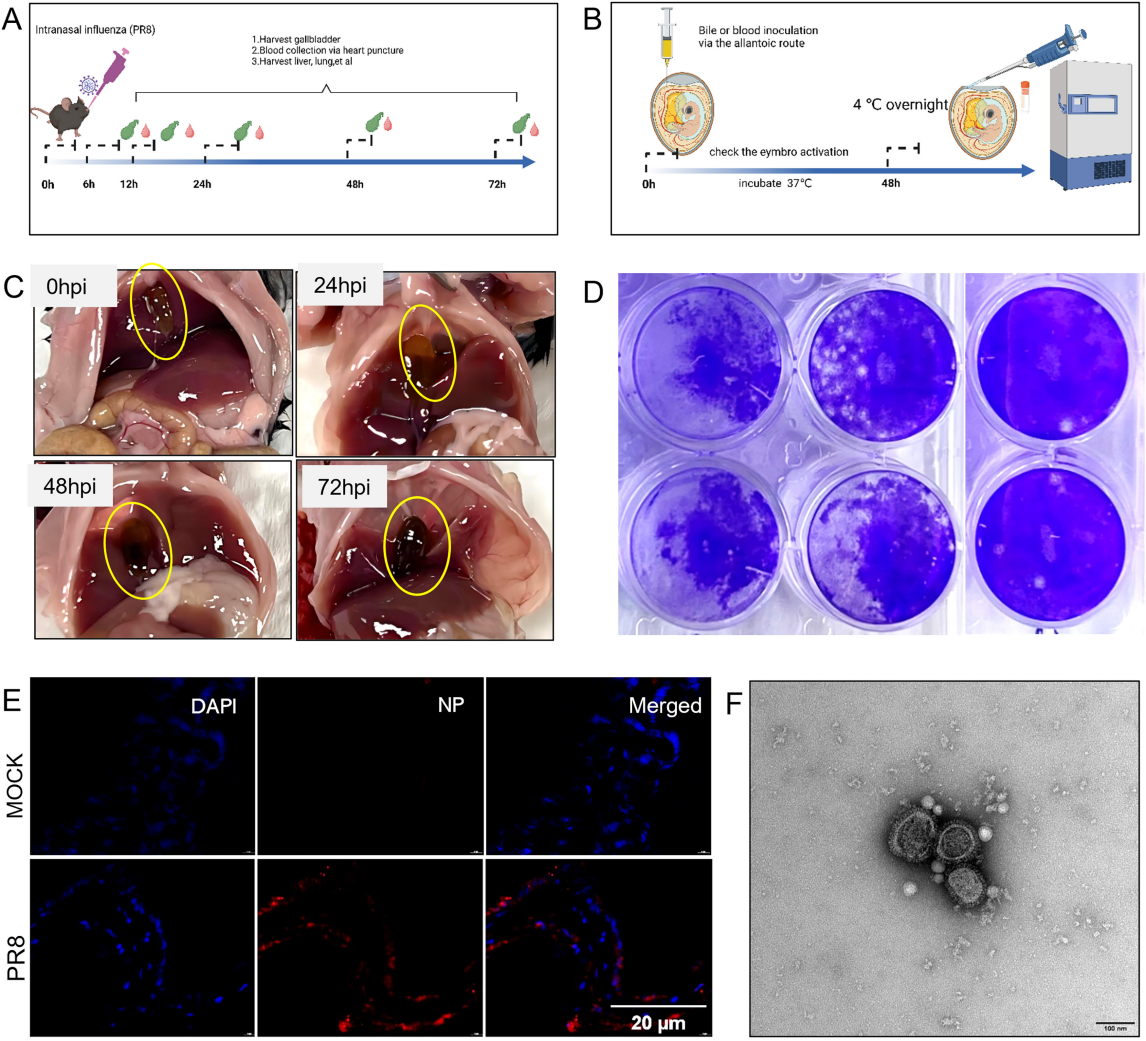
**Detection of influenza A (H1N1) virus in the bile of mice post intra‐nasal infection.** (A) Schematic diagram of animal experiment. (B) Schematic diagram of influenza virus propagation in embryonated chicken eggs. (C) The gallbladders of mice infected with PR8 at different times compared with mock (non‐infected) mouse. (D) After the inoculation of bile in embryonated chicken eggs, the virus titers in the cultured medium were measured by plaque assay. (E) Immunofluorescence staining of gall bladder sections of mock and PR8 infection mice. Red: NP protein of influenza A (H1N1) virus; Blue: DAPI for nuclei. (F) Negative stain electron microscopy showing the influenza virus in the incubation of bile acid in embryonated chicken eggs.

We first established a mouse model for severe influenza by infecting 12‐week‐old C57BL/6 mice with A/H1N1/Puerto Rico/8/34 virus via intra‐nasal route (Figure 1A). We observed that the gallbladder was swollen and inflamed after infection, especially at 48–72 h post infection (Figure 1C). Bile and peripheral blood samples were collected at different timepoints post infection: 6 h, 12 h, 24 h, 48 h, and 72 h and inoculated into embryonated eggs to detect the presence of live influenza virus (Figure 1B). We observed that live influenza virus can be cultured from bile sample at as early as 6 h post‐infection (Figure 1D). We also tested with the WSN strain virus in C57BL/6 mice and PR8 strain virus in BALB/c mice and identified similar phenotypes (Figure S2A). Fluorescent microscopy also revealed detection of influenza viral components in the gall‐bladder tissue at 24 h post infection (Figure 1E).

### 3.2 Influenza Virus Infection Leads to Transient Viremia and Liver Injury

**FIGURE 2:**
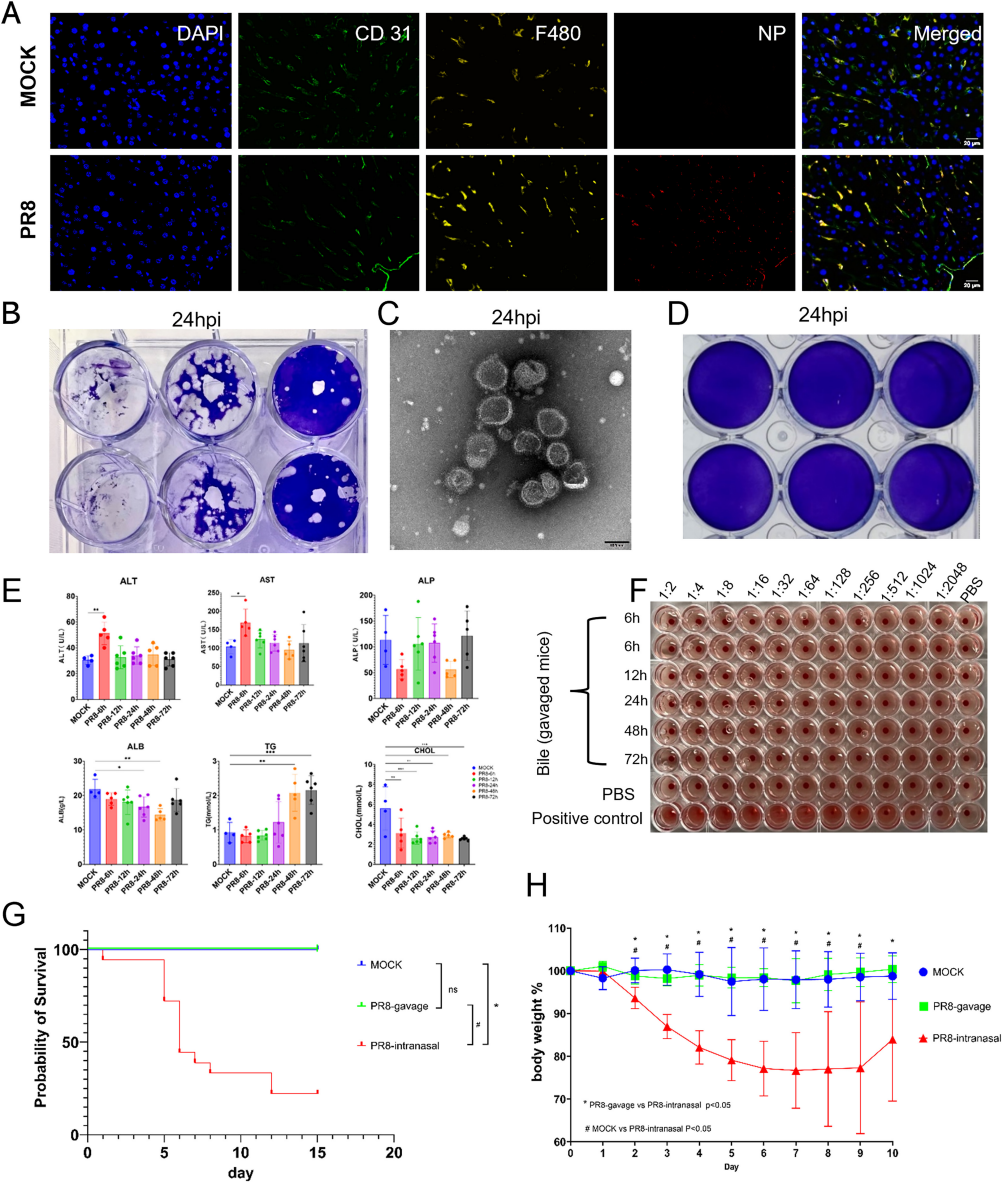
**Detection of transient influenza viremia and liver damage post infection.** (A) Immunofluorescence staining of liver sections of mock and PR8 infection (24 h) mice. Red: NP protein of influenza A (H1N1) virus; Blue: DAPI for nuclei; Green: CD31‐endothelial cells; Yellow: F480‐Kupffer cells. (B) The blood of severe influenza A (H1N1/PR8) virus infection mice was inoculated into embryonated chicken eggs for 48 h. The virus titer in the allontoic fluid was measured by plaque assay. (C) Negative stain electron microscopy showing the influenza virus in the blood sample of severe flu mice after propagating in embryonated chicken eggs. (D) The plaque assay of cultured medium of influenza virus gavage control group mice. (E) Serum biochemical markers related to liver function at different infection time. (F) The cultured medium of bile acids from the influenza virus gavage control group mice were quantified by hemagglutination assay with chicken erythrocyte. (G) Survival curve of mice. Blue: mock mice, treated with intranasal and gavage PBS; Green: PR8‐gavage mice, mice were gavaged with 1 × 10^4^ PFU influenza A(H1N1/PR8) virus; Red: severe influenza A (H1N1/PR8) virus infection mice, mice were infected intranasal with 1 × 10^6^ PFU influenza A (H1N1/PR8) virus. Survival data were plotted using the Kaplan–Meier survival curve. H) Body weights were monitored for 10 days and plotted using Prism software.

To further investigate the origin of the bile influenza virus, we collected the liver samples and stained for viral components. We observed that at 24 h post‐infection, the influenza virus NP protein can be detected in the liver tissue, and it co‐localized with Kupffer cells other than liver blood sinus endothelial cells (Figure 2A). The liver injury biomarkers (ALT and AST) were transiently elevated at 6 h post infection (Figure 2E). We further confirmed that live influenza virus was also detected in peripheral blood sample (Figure 2B,C). To exclude the possibility that the virus was spread into the liver in retrograde through the bile duct from the gastrointestinal tract, we gavaged the mice with influenza virus and collected the bile sample, but we did not detect live virus in bile from 6 to 72 h post‐infection (Figure 2D,F). The body weight of the gavaged mice did not decline post‐treatment. The liver pathology of the PR8 infected mice at different timepoints showed scattered areas with destruction of the hepatic lobule structure, cells with pyknotic nuclei and increased inter‐cellular space compared to the control mice. The cellular space also increased gradually with time after influenza infection. The PAS‐staining of liver tissues in PR8 infected mice demonstrated glycogen loss through time (Figure S2B).

### 3.3 Transcriptomic Change in the Liver After Influenza Virus Infection

**FIGURE 3:**
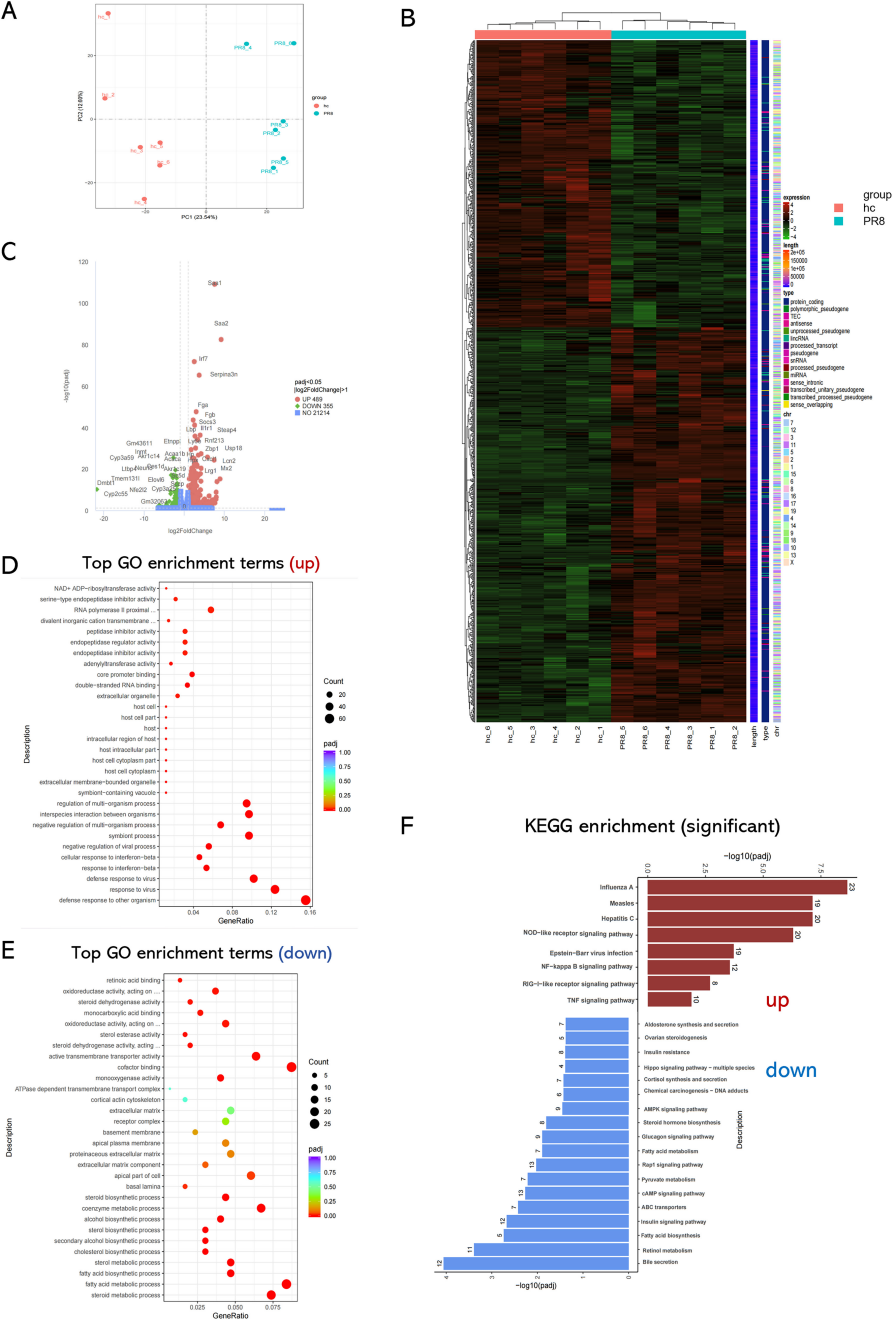
**The overview of the transcriptomic changes of mouse liver.** (A) PC biplot of transcriptome. (B) HC group and PR8 group difference gene clustering analysis heatmap. (C) Volcano plot of differential gene expression. (D–E) GO enrichment analysis: pathways up‐regulated (D) and down‐regulated (E) in severe influenza A (H1N1) infection mice group (PR8). (F) KEGG enrichment analysis of DEGs. Red indicates the upregulated pathways, blue indicates otherwise.

We tested the liver tissues for transcriptomic analysis. Influenza PR8 infection substantially changed the liver transcriptome at 24‐h post infection (Figure 3A,B). There are 489 genes up‐regulated and 355 genes down‐regulated in the influenza infection group compared with the control group. Among the top up‐regulated genes are acute phase protein SAA (serum amyloid A) and fibrinogen (Fga and Fgb), as well as inflammatory signal transducer IRF7 (interferon regulatory factor 7). Among the down‐regulated genes are biosynthetic process related genes, especially mapping to the bile secretion pathway (Figure 3E,F). The leading up‐regulated pathways of GO enrichment (Figure 3D) were mainly virus infection related pathways. The leading down‐regulated pathways of GO enrichment (Figure  3E) were mainly pathways related to liver function, including fatty acid metabolic and biosynthetic process, steroid and sterol metabolic process, coenzyme metabolic process, cholesterol biosynthetic process, and others. The findings were similar in the KEGG pathway enrichment analysis (Figure 3F).

### 3.4 Influenza Virus Infection Changes Bile Acid Metabolism in Liver

**FIGURE 4:**
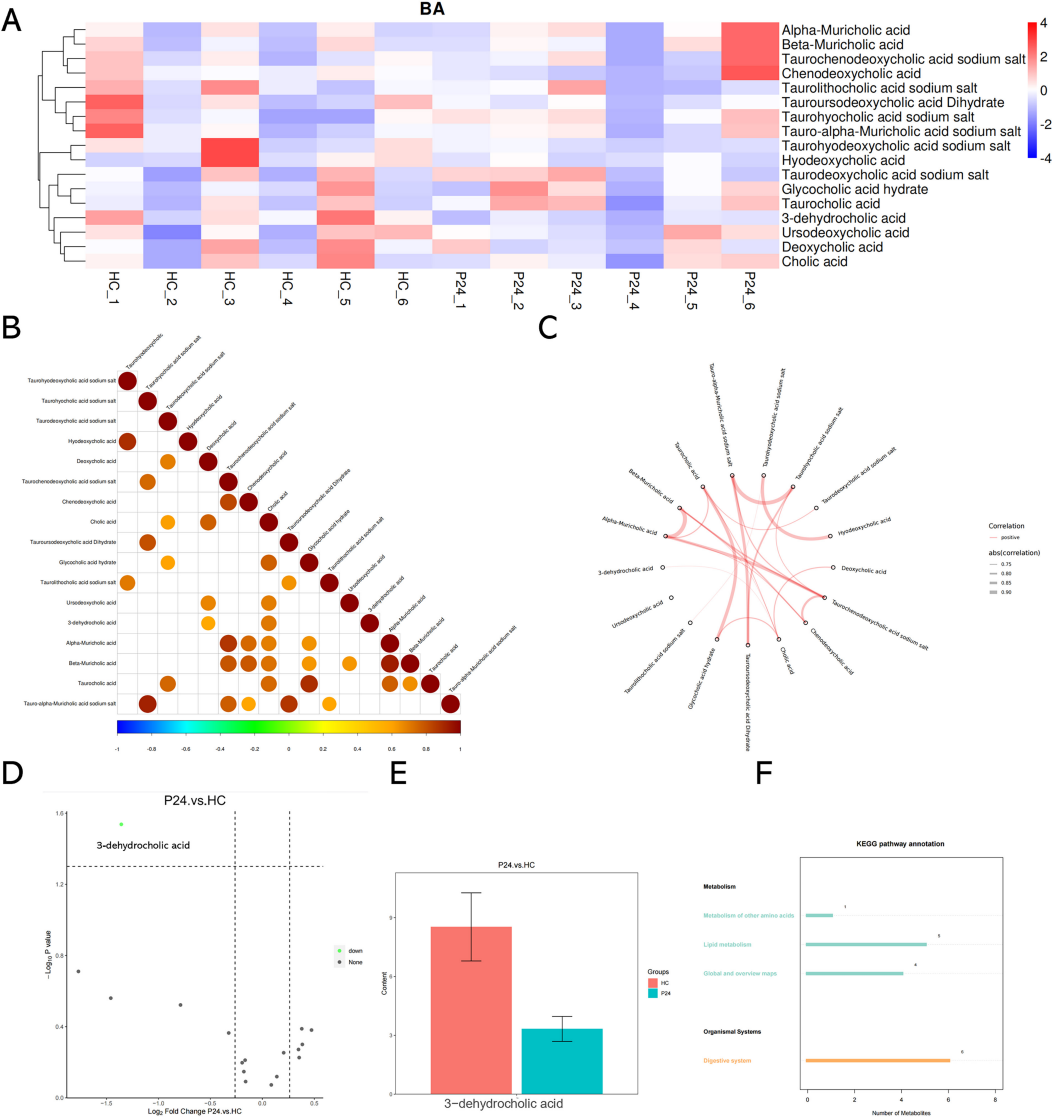
**Targeted metabolomic analyses of liver tissue for bile acid.** (A) Differential heatmap of bile acids in the liver tissue of mock (HC) and PR8‐infected mice according to targeted metabolomics analysis (*n* = 6 per group). The heatmap scale ranges from −4 to 4 on a log2 scale. (B) Correlation matrix of the bile acids. Significance statistical tests are conducted on metabolite correlation analysis with a threshold set at *p*‐value<0.05. (C) Correlation chord chart showing the correlations between different kinds of bile acids. The thickness of the lines connecting metabolites represents the magnitude of their correlation, with blue lines indicating negative correlations and red lines indicating positive correlations (only relationships between substances with absolute correlation coefficients greater than 0.7 are displayed). (D) Volcano maps of differential bile acids between severe influenza A infection (P24) and mock (HC) groups. Each volcanic map point represents a kind of bile acid; Abscissa, compare the logarithm of the number fold change of metabolites in the sample; Ordinate, the negative logarithm of *p*‐value. Increased abscissa absolute values indicate increased differences in expression multiples between the two samples, and increased ordinate values indicate increased significance in differential expression; green indicates down, gray indicates no significant difference. (E) Boxplot shows the bile acid that have significant difference between mock (HC) and severe influenza A(P24) groups. (F) KEGG enrichment diagrams of targeted metabolomic analyses of bile acids.

Influenza virus, as other membranous viruses, is vulnerable to bile acids. The detection of viable influenza particles in the bile suggests that the components of the bile may have changed so that influenza virus particle can survive. To test this hypothesis, we tested the liver tissues from control mice and PR8‐infected mice (24 h post infection) for targeted metabolomic analyses on the bile acid components (Figure 4A). In general, the bile acid metabolism pattern is variable across individual mice, yet one of the secondary bile acid metabolites (3‐dehydrocholic acid) was found to be universally down‐regulated in the PR8‐infected mice at 24‐h post infection (Figure 4D,E). The expression level of this metabolite is also positively correlated with the level of cholic acid (Figure 4B,C).

### 3.5 3‐Dehydrocholic Acid Protects Mice From Influenza

These observations of bile acid metabolism in liver and gut microbiota led us to focus on the role of bile acid in resistance to influenza virus infection, especially the 3‐dehydrocholic acid. We hypothesized that 3‐dehydrocholic acid may play an important role in host resistance to viral infection. To address this, we treated mice with 0.5 mM 3‐dehydrocholic acid in drinking water for entire duration of the experiments. After infection with influenza virus, mice that had been given 3‐dehydrocholic acid in drinking water for 7 days prior to infection had significantly decreased mortality and body weight loss compared with control mice (Figure S3A,B). The virus could not be detected in the bile of 3‐dehydrocholic acid treated mice with droplet digital PCR at 24 h post infection (Figure S3C).

Since bile acids are digestive surfactants, so they may disrupt influenza virus by destroying membrane integrity. PR8 influenza virus was treated with various concentration of 3‐dehydrocholic acid at 37 °C for 1 h and then titrated with plaque assays. We found that 3‐dehydrocholic acid inactivated the influenza virus at 20uM (Figure S3D).

### 3.6 Gut Microbiota was Reshaped After Influenza Virus Infection

We conducted sequencing of the 16S rRNA gene in the colon contents. Notably, significant disparities were observed in the gut microbiota composition between the PR8‐infected group and the healthy control (HC) group at both the genus and species levels (Figure S1A). The analysis identified 468 Operational Taxonomic Units (OTUs) common to both groups, alongside 41 and 43 unique OTUs in the PR8 and HC groups, respectively (Figure S2B). This finding corroborates previous research, suggesting that PR8 infection alters the gut microbiota composition [16].

Subsequently, we examined the 35 most abundant taxa at the species level in both groups and generated a heatmap. The analysis revealed marked differences in the abundance of *Lactobacillus murinus* , *Lactobacillus johnsonii* , and *Lactobacillus reuteri* between the HC and PR8 groups (Figure S1C). This is consistent with reports of a positive correlation between the genus *Lactobacillus* and 3‐Oxocholic Acid, supporting our observations [17]. We further analyzed the 100 most abundant genera in the population and constructed a phylogenetic tree to reinforce this association (Figure S1D). Additionally, the genera *Ligilactobacillus* and *Limosilactobacillus* exhibited variations between the PR8 and HC groups (Figure S1D). Our LEfSe analysis and the resulting Cladogram, along with the Linear Discriminant Analysis (LDA) scores from LEfSe, echoed these findings (Figures S1E and S1F). A subsequent t‐test analysis further substantiated our observations (Figures S1G and S1H).

## 4 Discussion

In this study, we detected live influenza virus in the bile in severe influenza mice early after intra‐nasal infection. We also detected transient viremia for influenza virus, and viral components in the liver co‐localizing with Kupffer cells. This indicates that the virus may have travelled from the origin of infection (the lungs) and spread through the blood stream to the liver and phagocytosed by the Kupffer cells (Figure 2A). To exclude the possibility that the virus travelled from the gastrointestinal tract backwards to the liver through the bile duct, we gavaged the mice with influenza virus, but we did not observe weight loss after infection or influenza virus detection in the bile system.

Immunopathology plays an important role in the pathogenesis of severe influenza. Respiratory viral sepsis (RVS) has been proposed recently to describe the situation of systemic damage and life‐threatening organ damage after infection with respiratory viruses including influenza [3, 18]. This has also been widely accepted for severe SARS‐CoV‐2 [19]. However, unlike the traditional bacterial sepsis where bacteremia is the key for diagnosis, there has been few evidence on the detection of viremia in respiratory viral sepsis, although detection of viral RNA and other components have been reported (Yan Y. et al, manuscript in submission, personal communication). Our study offers evidence for transient influenza viremia after respiratory infection. The liver may end up being the place for clearing the circulating virus, yet more evidence is needed. The mechanism for influenza virus passing the blood‐gas barrier is also intriguing. The relatively early detection of viremia suggests direct entrance of influenza virus possibly through damage of the epithelial or endothelial surface, rather than daughter viruses released into the circulation after replication in the epithelial cells. Further research is necessary for more detailed characterization.

The effect of influenza virus infection on liver injury and bile acid metabolism has not been well characterized. Our pathological data clearly shows damage to the liver cells after influenza infection (Figure S2). Bile acids and the intestinal microbiome exhibit a close relationship [20]. Previous studies have indicated that influenza infection can modify the gut microbiota composition, which, in turn, may influence the host's resistance to the influenza virus [21]. Based on these observations, we proposed that influenza infection induces alterations in the gut microbiota pertinent to bile acid production. Indeed, sequencing of the 16S rRNA gene in colon contents has unveiled significant shifts in the gut microbiota due to influenza infection, particularly affecting bile acid metabolism. The genus *Lactobacillus*, associated with 3‐Oxocholic Acid, along with other significantly altered species and genera, have been identified as key players in bile acid metabolism [17, 22, 23].

An increasing body of research suggests that the composition of gut microbiota, in conjunction with the host's regulation of bile acid transport and synthesis, constitutes the bile acid pool [20]. In this study, we performed 16S rRNA sequencing on the colonic contents of mice subjected to influenza virus infection, aiming to pinpoint specific gut microbiota involved in the modulation of secondary bile acid formation in the context of influenza virus infection. This effort seeks to shed light on the modifications observed in secondary bile acids within metabolomic profiles. At the genus level, significant disparities were observed in *Ligilactobacillus*, *Limosilactobacillus*, and *Lactobacillus* between the Healthy Control (HC) and the influenza infected (PR8) groups. Prior research indicates that a reduction in *Ligilactobacillus* is associated with decreased bile acid concentrations in the gut [24]. In the scenario of a diet mimicking fast food consumption, *Limosilactobacillus reuteri* was found to enhance glucoregulatory functions, mitigate the progression of non‐alcoholic fatty liver disease (NAFLD), and elevate distal gut bile acid levels [23]. Moreover, *Limosilactobacillus reuteri* has been shown to modify bile acid composition in mice against the backdrop of cholesterol gallstones [25]. Within the *Lactobacillus* genus, we identified three species— *L. murinus* , *L. johnsonii* , and *L. reuteri* —exhibiting statistically significant differences between the HC and PR8 groups. *L. johnsonii* has been closely linked to differential biomarkers associated with the pathogenesis and advancement of cholestasis in the context of FXR knockout [26]. *L. reuteri* NCIMB 30242, known for its bile salt hydrolase activity, has been demonstrated to elevate circulating bile acids and significantly increase total, conjugated, and unconjugated bile acids in participants of a clinical study [27]. Additionally, extensive animal research has corroborated that *L. reuteri* mitigates liver inflammation by modulating bile acid metabolism [28, 29]. Synthesizing the insights from prior studies with our findings, we propose a novel mechanism by which the gut microbiome influences influenza infection. Influenza infection alters the gut microbiome composition, which subsequently modifies bile acid metabolism, diminishing the bile's capacity to deactivate the influenza virus. This impairment allows the virus to persist within the biliary system, thereby intensifying viral sepsis. While current investigations into the interplay between the gut microbiome and the host during respiratory infections have predominantly concentrated on inflammation and immunity [30, 31], our exploration of bile acid metabolism introduces an entirely novel avenue of research.

Our study has several limitations. Firstly, this phenotype is described in mice model, and its clinical significance still awaits verification. Collecting bile sample from patients with acute influenza virus infection is challenging and bears ethical problems. Secondly, we did not fully characterize the mechanism for influenza virus to enter the blood stream and cleared in the liver.

In conclusion, we reported that influenza virus infection leads to early transient viremia and the virus particle can be detected in the bile sample. Influenza infection also alters the liver metabolism pathways. This offers potential evidence for respiratory viral sepsis.

## Author Contributions

**Yan Liu:** conceptualization, methodology, software, data curation, supervision, formal analysis, validation, investigation, writing – original draft, project administration, visualization, resources. **Jiuyang Xu:** conceptualization, writing – original draft, writing – review and editing, supervision. **Cheng Wei:** software. **Yitian Xu:** methodology. **Chen Lyu:** writing – original draft. **Mingzhi Sun:** methodology. **Ying Zheng:** data curation, methodology. **Bin Cao:** funding acquisition, conceptualization, writing – review and editing, project administration.

## Ethics Statement

The animal study protocol was approved by the Ethics Committee of Beijing Animal Research Center Co. Ltd (protocol code BLARC‐JSB‐DW/013‐JL/001, 2022‐10‐20).

## Conflicts of Interest

The authors declare no conflicts of interest. The funders had no role in the design of the study; in the collection, analyses, or interpretation of data; in the writing of the manuscript; or in the decision to publish the results.

### Peer Review

The peer review history for this article is available at https://www.webofscience.com/api/gateway/wos/peer‐review/10.1111/irv.70012.

## Supporting information


**Figure S1** Gut microbiota was reshaped in sever influenza A(H1N1) infected mice.
**Figure S2.** Live H1N1 influenza virus detected in the blood and bile from several mice model.
**Figure S3.** 3‐dehydrocholic acid protects mice from influenza.

## Data Availability

The original contributions presented in the study are included in the article/supplementary material; further inquiries can be directed to the corresponding author/s.
