# Supplementary material for: Detection of H1N1 Influenza Virus in the Bile of a Severe Influenza Mouse Model
Source: Influenza Other Respir Viruses. 2024 Oct 25;18(10):e70012. doi: 10.1111/irv.70012 (PMC11502934; doi:10.1111/irv.70012)
Supplement: Supplementary file 1 — Figure S1 Gut microbiota was reshaped in sever influenza A(H1N1) infected mice. Figure S2. Live H1N1 influenza virus detected in the blood and bile from several mice model. Figure S3. 3‐dehydrocholic acid protects mice from influenza. [file IRV-18-e70012-s001.doc]

**Supplementary Material**

**Detection of H1N1 Influenza Virus in the Bile of a Severe Influenza Mouse Model**

Yan Liu^1,2^, Jiuyang Xu^1^, Cheng Wei^3^, Yitian Xu^4^, Chen Lyu^4^, Mingzhi Sun^5^, Ying Zheng^6^, and Bin Cao^1^

^1^ National Center for Respiratory Medicine; State Key Laboratory of Respiratory Health and Multimorbidity; National Clinical Research Center for Respiratory Diseases; Institute of Respiratory Medicine, Chinese Academy of Medical Sciences; Department of Pulmonary and Critical Care Medicine, Center of Respiratory Medicine, China-Japan Friendship Hospital, Beijing, China

^2^ Department of Critical Care Medicine, Yantai Affiliated Hospital of Binzhou Medical University, Yantai 264100, Shandong, China

^3^ Peking University China-Japan Friendship School of Clinical Medicine, Beijing, China

^4^ Peking Union Medical College and Chinese Academy of Medical Sciences, Beijing, China

^5^ Tsinghua University School of Medicine, Beijing, China

^6^ Department of Pulmonary and Critical Care Medicine, China-Japan Friendship hospital, Capital Medical University, Beijing, China

Correspondence: Prof. Bin Cao ([caobin_ben@163.com](mailto:caobin_ben@163.com))

**Table of contents**

Figure S1 Gut microbiota was reshaped in sever influenza A(H1N1) infected mice………………….2

Figure S2 Live H1N1 influenza virus detected in the blood and bile from several mice model………4

Figure S3 3-dehydrocholic acid protects mice from influenza…………………………………………5

Supplementary Methods ……………………………………………………………………………….6

**Supplementary Figures**


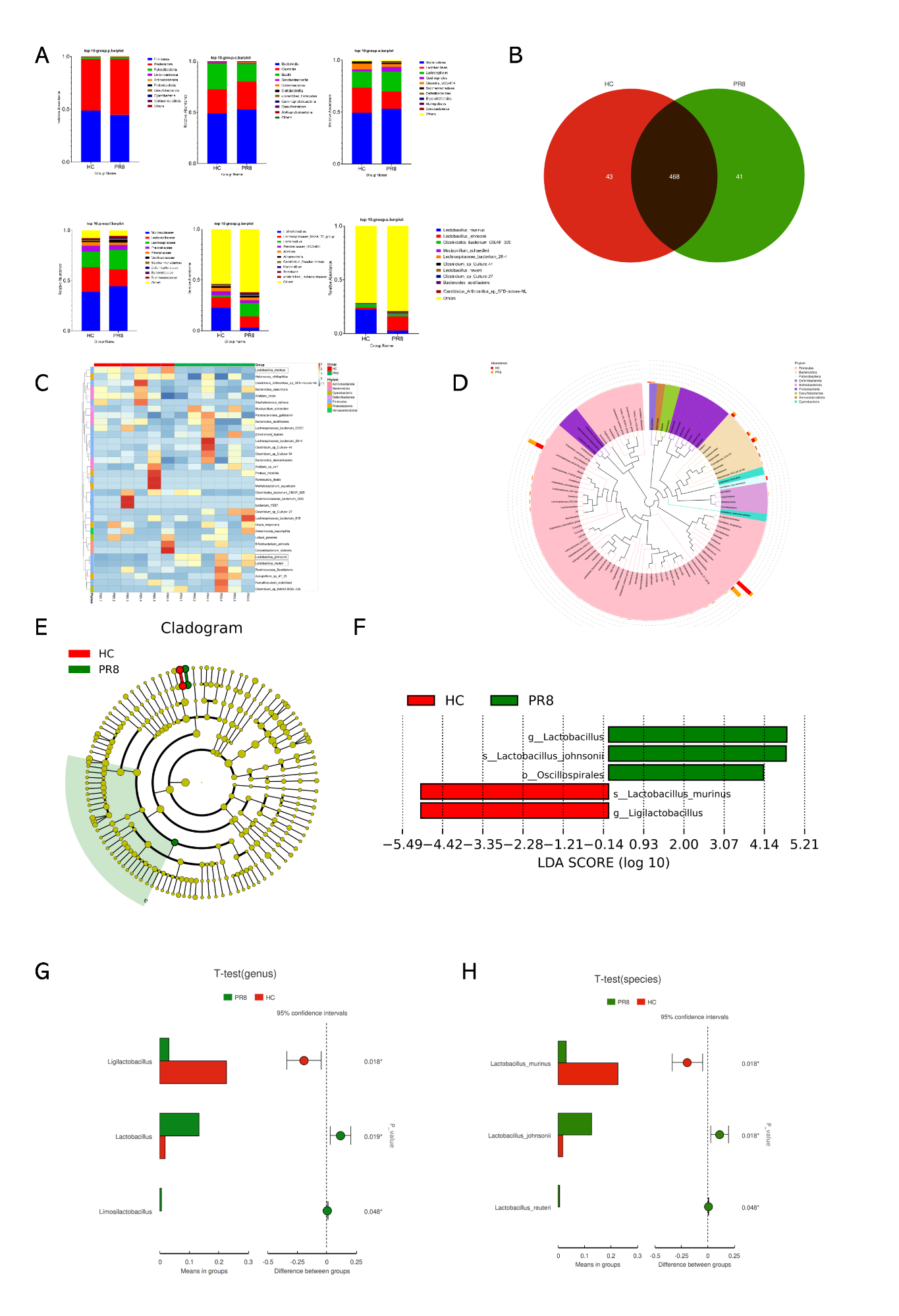


**Figure S1 Gut microbiota was reshaped in sever influenza A(H1N1) infected mice.**

A) Compositional profile of murine gut microbiota at different levels (phylum, class, order, family, genus and species). B) Venn diagrams visually display the common and unique information between different samples or groups. C) Heatmap diagrams of top 35 taxa of each sample at phylum level. The scale was from -1 to 3. The black boxes show the phylum that had significant difference. D) Phylogenetic tree of top 100 genus. One hundred genera with the highest abundance in the groups were selected and performed sequence alignment to draw the phylogenetic tree in Perl with SVG function. E) Cladogram of LEfSe analysis. F) LEfSe linear discriminant analysis (LDA) scores. Bacterial taxa identified as differentially abundant between the healthy control (PBS treatment) and the severe influenza A infection group (PR8 treatment) by LEfSe. Green indicates bacterial taxa whose abundance was higher in the PR8 group; red indicates otherwise. Significantly abundant gut microbial taxa at the genus(G) and species(H) levels between two groups, identified using t-test analysis. otherwise. (G-H) Significantly abundant gut microbial taxa at the genus(G) and species(H) levels between two groups, identified using t-test analysis.


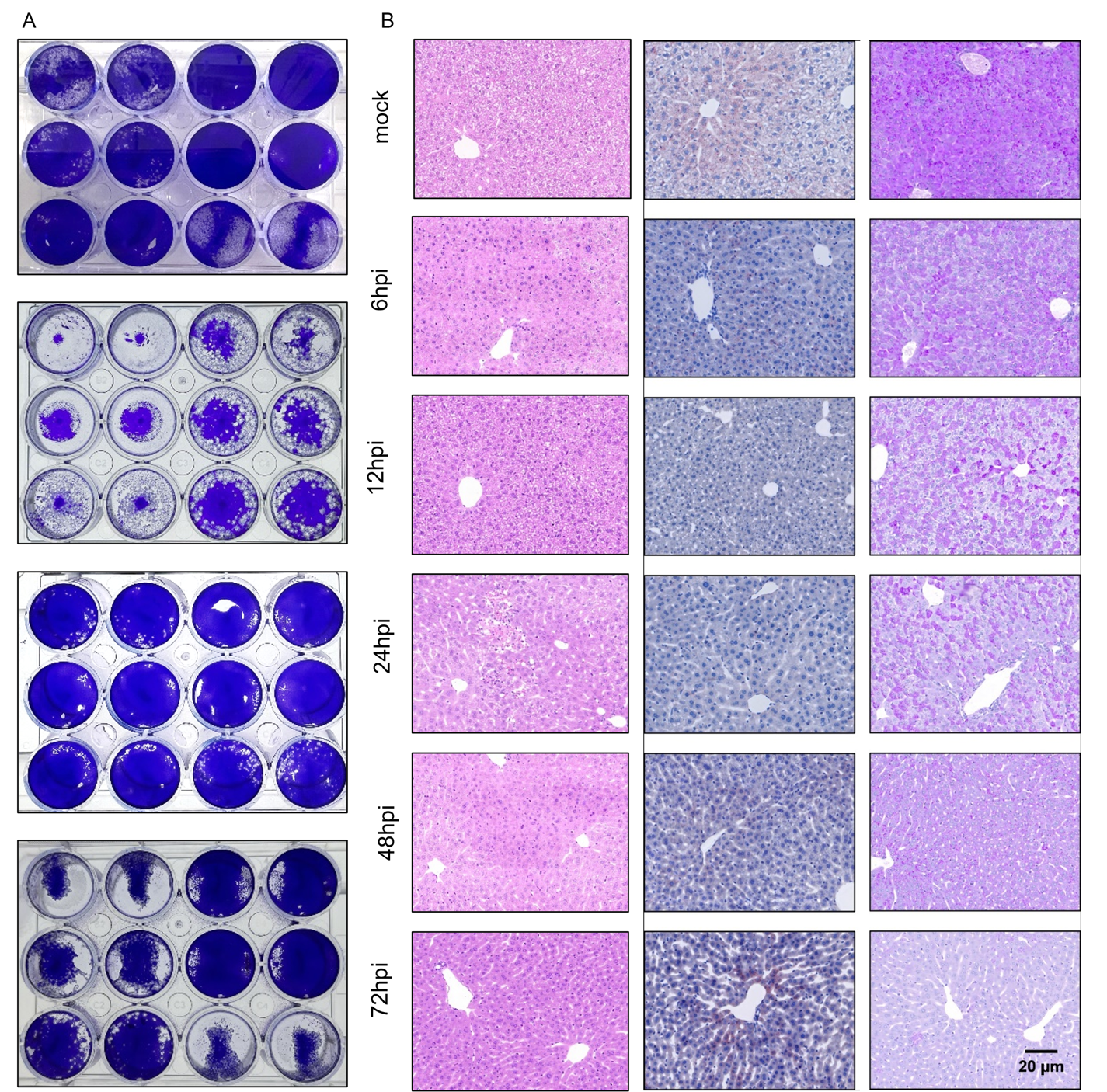


**Figure S2 Live H1N1 influenza virus was detected in the blood and bile in several mouse model**

A) Blood and bile samples collected from WSN-intranasal-infected C57BL/6 and PR8-intranasal-infected Balb/c mice were incubated in embryonated chicken eggs, the virus titers in the cultured medium were measured by plaque assay (from up to down: blood cultured medium from WSN-intranasal-infected mice, bile cultured medium from WSN-intranasal-infected mice, blood cultured medium from PR8-intranasal-infected Balb/c mice, bile cultured medium from PR8-intranasal-infected mice); B) Representative results from mouse liver tissue sections stained with H&E, Oil red O, and PAS stain. Scale bar, 20 µm.


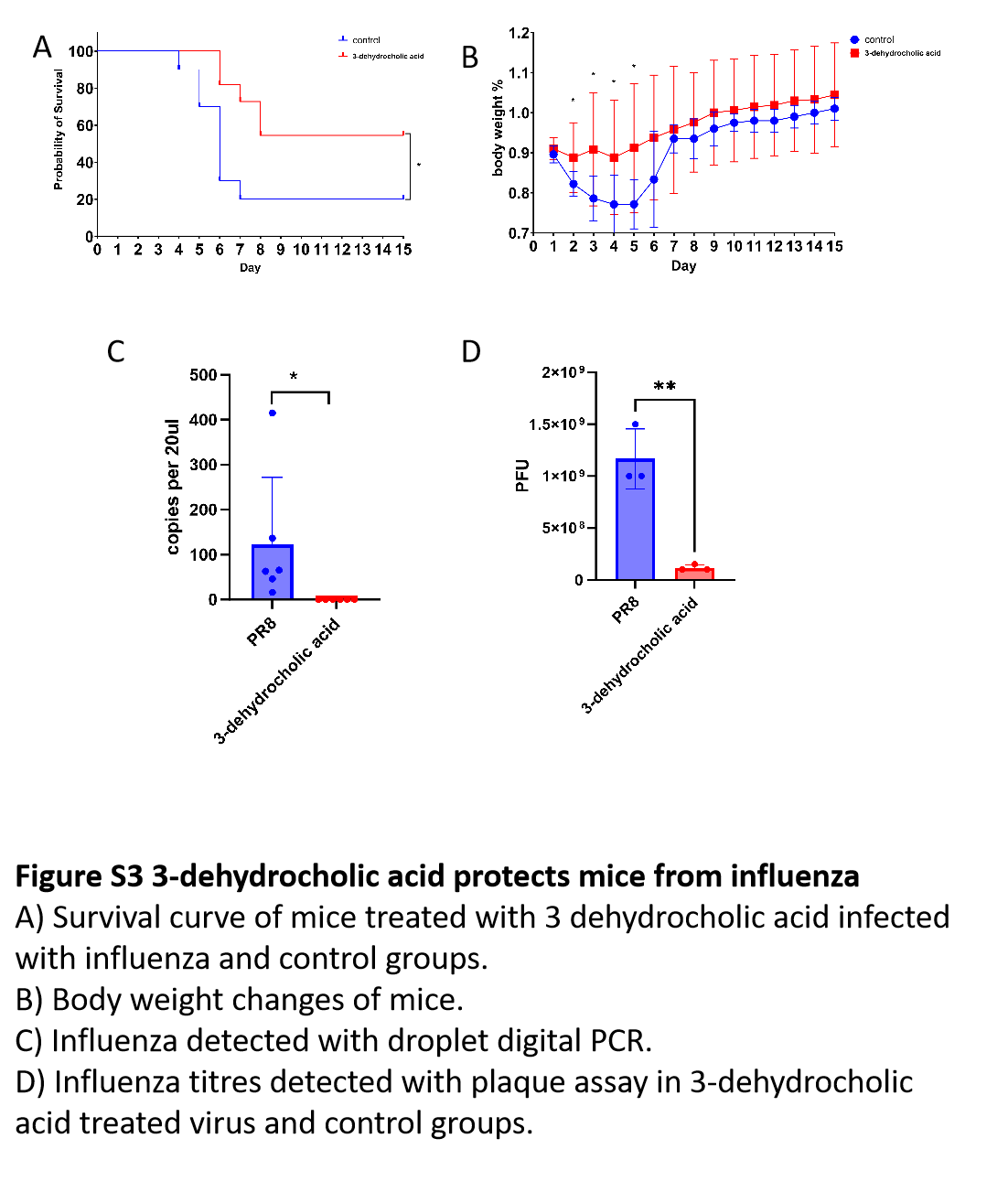


**Figure S3 3-dehydrocholic acid protects mice from influenza**

A) Survival curve of mice treated with 3 dehydrocholic acid infected with influenza and control groups. B) Body weight changes of mice. C) Influenza detected in bile with droplet digital PCR. D) Influenza titres detected with plaque assay in 3-dehydrocholic acid treated virus and control groups.

**Supplementary Methods**

*Bulk RNA sequencing*

*RNA extraction, library preparation and sequencing.* Liver samples of mice were harvested and total RNA was extracted with TRIzol reagent (Thermo Fisher Scientific, USA). Then, high-throughput RNA-sequencing was performed by Novogene Technology (Beijing, China). RNA integrity was assessed using the RNA Nano 6000 Assay Kit of the Bio analyzer 2100 system (Agilent Technologies, CA, USA). Total RNA was used as input material. Briefly, mRNA was purified from total RNA using poly-T oligo-attached magnetic beads. Fragmentation was carried out using divalent cations under elevated temperature in First Strand Synthesis Reaction Buffer(5X). First strand cDNA was synthesized using random hexamer primer and M-MuLV Reverse Transcriptase, then use RNaseH to degrade the RNA. Second strand cDNA synthesis was subsequently performed using DNA Polymerase I and dNTP. Remaining overhangs were converted into blunt ends via exonuclease/polymerase activities. After adenylation of 3’ ends of DNA fragments, Adaptor with hairpin loop structure were ligated to prepare for hybridization. In order to select cDNA fragments of preferentially 370~420 bp in length, the library fragments were purified with AMPure XP system (Beckman Coulter, Beverly, USA). Then PCR was performed with Phusion High-Fidelity DNA polymerase, Universal PCR primers and Index (X) Primer. At last, PCR products were purified (AMPure XP system) and library quality was assessed on the Agilent Bioanalyzer 2100 system. The clustering of the index-coded samples was performed on a cBot Cluster Generation System using TruSeq PE Cluster Kit v3-cBot-HS (Illumia) according to the manufacturer’s instructions. After cluster generation, the library preparations were sequenced on an Illumina Novaseq platform and 150 bp paired-end reads were generated.

*Transcriptome analysis.* Raw data were firstly removed reads containing adapter, containing N base and low-quality reads to get the clean data. At the same time, Q20, Q30 and GC content the clean data were calculated. Reference genome and gene model annotation files were downloaded from genome website directly. Index of the reference genome was bult using Hisat2 v2.0.5 and paired-end clean reads were aligned to the reference genome using Hisat2 v2.0.5. FeatureCounts v1.5.0-p3 was used to count the reads numbers mapped to each gene. And then Fragments per Kilobase of transcript sequence per Millions (FPKM) of each gene was calculated based on the length of the gene and reads count mapped to this gene. For each sequenced library, the read counts were adjusted by edge R program package through one scaling normalized factor. Differential expression analysis of two groups was performed using the edgeR R package (3.22.5). The P values were adjusted using the Benjamini & Hochberg method. Corrected P-value of 0.05 and absolute fold change of 2 were set as the threshold for significantly differential expression.Then, differential expression analysis of mock (HC) and PR8 groups was performed using the DESeq2 R package[1, 2] (1.20.0). DESeq2 provide statistical routines for determining differential expression in digital gene expression data using a model based on the negative binomial distribution. The resulting P-values were adjusted as above. Genes with an adjusted P-value <0.05 found by DESeq2 were assigned as differentially expressed. Gene Ontology (GO) and KEGG enrichment analysis of differentially expressed gene was implemented by the clusterProfilter R package[3], in which gene length bias was corrected. The threshold of p value was 0.05. The Benjamini-Hochbergch procedure was used to adjust the p value. Gene set enrichment analysis (GSEA) was performed using clusterProfiler v 4.10.0 and visualized by R package GseaVis v 0.1.0.

*Bile acid metabolomic*

*Standard solution preparation.* The stock solution of individual bile acid was mixed and prepared in bile acid-free matrix to obtain a series of bile acid calibrators at a concentration of 25000, 15000, 5000, 2500, 500, 250, 50, 25, 15, 5, 2.5 or 1.5 ng/mL. Certain concentrations of GCA-d4, UDCA-d4, CA-d4, GCDCA-d4, LCA-d4 and CDCA-d4 were compounded and mixed as Internal Standard (IS). The stock solution of all of these and working solution were stored in refrigerator of -20°C.

*Metabolites extraction.* The samples of liver tissue were resuspended with liquid nitrogen, and then added to water by well vortexing as the diluted sample. Then 100 μL of them were taken respectively and homogenized with 300 μL of acetonitrile/methanol (8:2) which contained mixed internal standards by well vortexing. Next, put it on ice for 30min. After that, centrifuged at 12,000 rpm for 10 min. Finally, the supernatant was injected into the LC-MS/MS system for analysis.

*LC-MS method.* An ultra-high performance liquid chromatography coupled to tandem mass spectrometry (UHPLC-MS/MS) system (ExionLC™ AD UHPLC-QTRAP 6500+, AB SCIEX Corp., Boston, MA, USA) was used to quantitate bile acids in Novogene Co., Ltd. (Beijing, China). Separation was performed on a Waters ACQUITY UPLC BEH C18 column (2.1×100mm, 1.7μm) which was maintained at 50°C. The mobile phase, consisting of 0.1% formic acid in water (solvent A) and acetonitrile (solvent B), was delivered at a flow rate of 0.30 mL/min. The solvent gradient was set as follows: initial 20% B, 0.5min; 20-35% B, 1min; 35-37% B, 2.5 min; 37-38% B, 4.1min; 38-39% B, 6min; 39-40% B, 6.5min; 40-44% B, 8.5min; 44-45% B, 9min; 45-52% B, 9.5min; 52-65% B, 12.5min; 65-100% B, 13min; 100-20% B, 15.1min; 20% B, 17 min. The mass spectrometer was operated in negative multiple reaction mode (MRM) mode. Parameters were as follows: IonSpray Voltage (-4500 V), Curtain Gas (35 psi), Ion Source Temp (550°C), Ion Source Gas of 1 and 2 (60 psi).

*Data acquisition and analysis*. Data acquisition and instrumental control were performed with Analyst 1.7 software (Sciex, Darmstadt, Germany). The data were analyzed with MultiQuant 3.0.3 (Sciex, Darmstadt, Germany) and Metaboanalyst [4].

*Gut microbiota analysis*

The colonic contents of mice were collected and frozen at -80℃. The 16s rRNA sequencing were performed by Novogene Technology (Beijing, China). DNA was extracted with DNA extraction kit (QIAamp DNA stool mini kit, Qiagen, Hilden, Germany) for real-time fluorescence quantitative polymerase chain reaction (PCR), which amplified the V3–V4 region of the bacterial 16s rRNA gene. The purified amplicons were sequenced by Illumina MiSeq system, and the high-quality sequences were clustered according to 97% similarity by de novo UCLUST algorithm to obtain operational taxonomic unit (OTU). Then, the α-diversity index was assessed, and the difference of OTUs was analyzed by Mann–Whitney nonparametric test. Linear discriminant analysis (LDA) effect size (LEfSe) analysis was used to identify differential marker species by LDA algorithm.

*Animals*

Mice were treated with 0.5mM of 3-dehydrocholic acid (MCE, HY-N7387) in drinking water 7 days before infection and continued for the entire duration of the experiments. Ten 6-week-old male C57 mice were used in this group, mice were intranasal infected with 10^3^ PFU PR8 influenza virus, then monitored daily for weight loss and survival for 15 days. Eleven mice with same age and sex were included in the control group. Kaplan-Meier survival curves were plotted for each group. The other 6 pairs of mice that treated with 3-dehydrocholic acid were used to observe virus quantification in the gallbladder, while 6 mice infected as above and the other mice were used as control.

*RNA extraction and expression assessment using digital droplet PCR (ddPCR)*

Total RNA was extracted from bile that collected from gallbladders using the RNeasy minikit (Qiagen) according to the manufacturer’s instructions. The ddPCR was performed following the manufacturer’s protocol using One-Step RT ddPCR Adv Kit 200 rxns (Bio-Rad, USA), a DG8 Cartridges for QX200 droplet generator (Bio-Rad), a QX200 droplet reader (Bio-Rad), and QuantaSoft software (Bio-Rad). The primers of NP gene of influenza A were: forward-GACCRATCCTGTCACCTCTGAC, reverse- GGGCATTYTGGACAAAKCGTCTACG, probe FAM-TGCAGTCCTCGCTCACTGGGCACG-BHQ1. Sterile, enzyme-free water was selected as the negative control.
